# A targeted metabolomics method for extra- and intracellular metabolite quantification covering the complete monolignol and lignan synthesis pathway

**DOI:** 10.1016/j.mec.2022.e00205

**Published:** 2022-08-31

**Authors:** Andrea Steinmann, Katrin Schullehner, Anna Kohl, Christina Dickmeis, Maurice Finger, Georg Hubmann, Guido Jach, Ulrich Commandeur, Marco Girhard, Vlada B. Urlacher, Stephan Lütz

**Affiliations:** a Chair for Bioprocess Engineering, TU Dortmund University, Emil-Figge-Straße 66, 44227, Dortmund, Germany; b Phytowelt GreenTechnologies GmbH, Kölsumer Weg 33, 41334, Nettetal, Germany; c Institute of Molecular Biotechnology, RWTH Aachen University, Kackertstraße 9, 52072, Aachen, Germany; d Institute of Biochemistry, Heinrich-Heine University, Universitätsstraße 1, 40225 Düsseldorf, Germany

**Keywords:** Lignans, Targeted metabolomics, Metabolite extraction, Metabolic engineering, Heterologous production, Method development

## Abstract

Microbial synthesis of monolignols and lignans from simple substrates is a promising alternative to plant extraction. Bottlenecks and byproduct formation during heterologous production require targeted metabolomics tools for pathway optimization.

In contrast to available fractional methods, we established a comprehensive targeted metabolomics method. It enables the quantification of 17 extra- and intracellular metabolites of the monolignol and lignan pathway, ranging from amino acids to pluviatolide. Several cell disruption methods were compared. Hot water extraction was best suited regarding monolignol and lignan stability as well as extraction efficacy. The method was applied to compare enzymes for alleviating bottlenecks during heterologous monolignol and lignan production in *E. coli*. Variants of tyrosine ammonia-lyase had a considerable influence on titers of subsequent metabolites. The choice of multicopper oxidase greatly affected the accumulation of lignans. Metabolite titers were monitored during batch fermentation of either monolignol or lignan-producing recombinant *E. coli* strains, demonstrating the dynamic accumulation of metabolites.

The new method enables efficient time-resolved targeted metabolomics of monolignol- and lignan-producing *E. coli*. It facilitates bottleneck identification and byproduct quantification, making it a valuable tool for further pathway engineering studies. This method will benefit the bioprocess development of biotransformation or fermentation approaches for microbial lignan production.

## Highlights

• Monolignols and lignans were heterologously produced in *Escherichia coli*
• A targeted metabolomics method was developed covering 17 out of 20 metabolites.
• Hot water extraction is well suited for intracellular monolignol & lignan analysis.
• Metabolite accumulation identifies bottlenecks and dynamic activity.
• Assessment of pathway activity enables efficient cell factory engineering.

## Abbreviations:

DAD: Diode array detector
PAL: Phenylalanine ammonia-lyase
TAL: Tyrosine ammonia-lyase
C4H: Cinnamate 4-hydroxylase
4HPA3H: 4-Hydroxyphenylacetate 3-monooxygenase
COMT: Caffeic acid O-methyltransferase
CCoAOMT: Caffeoyl-CoA O-methyltransferase
4CL: 4-Coumarate:CoA ligase
CCR: Cinnamoyl-CoA reductase
CAD: Cinnamyl-alcohol dehydrogenase
-[H]: One-electron oxidation
CgL1: Laccase from *Corynebacterium glutamicum*
CueO: Copper efflux oxidase
PLR: Pinoresinol-lariciresinol reductase
SDH: Secoisolariciresinol dehydrogenase
HClO_4_: Perchloric acid cell disruption
H_2_O: Hot water cell disruption
MeOH: Cold methanol cell disruption
KOH: Potassium hydroxide cell disruption
RI: Refractive index
C_intra_: Intracellular metabolite titer
c_HPLC_: Concentration measured during HPLC-DAD-MS analysis
V_solvent_: Volume of solvent used during cell disruption
V_sample_: Sample volume
SIV: Specific intracellular volume
Rs: *Rhodobacter sphaeroides*
Ha: *Herpetosiphon aurantiacus*
Rg: *Rhodotorula glutinis*

## 1 Introduction

Lignans are a diverse class of phenylpropanoid dimers that offer great potential for promoting human health (Teponno et al., 2016). Isolation from native plant producers suffers from drawbacks such as low titers, long growth cycles, or dependency on environmental conditions (Mark et al., 2019). Heterologous microbial production is desired to overcome these limitations. In a recent study, pluviatolide, a precursor of the anti-cancer drugs teniposide and etoposide, was produced heterologously with *Escherichia coli* (*E. coli*), using coniferyl alcohol as substrate (Decembrino et al., 2020). Heterologous de novo synthesis of coniferyl alcohol has already been achieved in *E. coli* (Chen et al., 2017). Thus, in theory, the complete whole-cell synthesis of pluviatolide from intracellular primary metabolites of *E. coli* or externally supplied simple substrates such as amino acids is feasible.

**Fig. 1:**
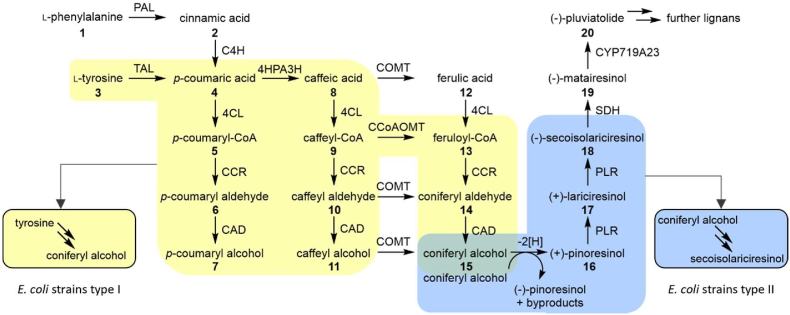
Synthesis pathway of monolignol and lignan metabolites from phenylalanine/tyrosine to pluviatolide. Pathway sections highlighted in color were studied in vivo. The yellow section is included in *E. coli* strains type I, which produce coniferyl alcohol from tyrosine. *E. coli* strains type II express the genes necessary for the pathway section highlighted in blue, resulting in the production of secoisolariciresinol from coniferyl alcohol. PAL: phenylalanine ammonia-lyase; TAL: tyrosine ammonia-lyase; C4H: cinnamate 4-hydroxylase; 4HPA3H: 4-hydroxyphenylacetate 3-monooxygenase; COMT: caffeic acid O-methyltransferase; CCoAOMT: caffeoyl-CoA O-methyltransferase; 4CL: 4-coumarate:CoA ligase; CCR: cinnamoyl-CoA reductase; CAD: cinnamyl-alcohol dehydrogenase; –[H]: one-electron oxidation, for instance, catalyzed by laccase from *Corynebacterium glutamicum* (*Cg*L1) or copper efflux oxidase (CueO); PLR: pinoresinol-lariciresinol reductase; SDH: secoisolariciresinol dehydrogenase; CYP: P450 monooxygenase. (For interpretation of the references to color in this figure legend, the reader is referred to the Web version of this article.)

A heterologous pathway of at least eleven steps is necessary to synthesize the complex lignan pluviatolide from primary metabolites supplied by the host (Fig. 1). Although *E. coli* is a fast-growing and undemanding host, several new challenges emerge when the new pathway is integrated in *E. coli*. Firstly, the promiscuity of the participating enzymes and the unavailability of protein glycosylation result in byproduct formation (Aschenbrenner et al., 2019; Pickel and Schaller, 2013). For example, laccases initiating the coupling of two coniferyl alcohol (**15**) units yield at least three coupling products and also accept the resulting target lignan pinoresinol (**16**) as substrate, leading to overoxidation and oligo-/polymerization (Tarrago et al., 2018). Secondly, low enzyme activities might lead to the accumulation of intermediates and bottleneck formation. For instance, the amount of carbon that is directed towards the heterologous product synthesis depends on the first step of the cascade, tyrosine (**3**) deamination catalyzed by a tyrosine ammonia-lyase (TAL) (Haslinger and Prather, 2020). However, most TALs exhibit low activity, resulting in rate limitation (Lin and Yan, 2012; Vannelli et al., 2007). Accumulation of metabolites potentially leads to enzyme inhibition and cell toxicity (Sariaslani, 2007). To prevent byproduct synthesis as well as accumulation of intermediates and to ensure an optimal flow of carbon from the substrate to the final heterologous product, enzyme activities of the various steps need to be carefully adjusted. For example, this can be achieved via the choice of enzyme variants, gene copy numbers, or promoter strengths (Jones and Koffas, 2016). The addressed challenges require quantification of all metabolite titers of the heterologous pathway.

Without separation, quantifying individual metabolites is not possible for complex mixtures of analytes, such as in samples from cultures producing monolignols and lignans. This is due to the overlapping UV absorption profiles of the various analytes (An et al., 2016). Thus, the main challenge in quantifying metabolites of the monolignol and lignan synthesis pathway is the separation of several analytes covering a relatively large range of hydrophilicity/-phobicity, while still allowing proper separation of metabolites with rather similar properties. Chromatographic methods are used to separate and detect various analytes in complex samples in a single run. Thin layer chromatography has previously been used to analyze plant-derived lignans but is not well suited for quantification (Slanina and Glatz, 2004). In contrast, coupling high-performance liquid chromatography (HPLC) or gas chromatography (GC) to mass spectrometry (MS) or diode array detectors (DAD) is feasible for quantification. HPLC is favored over GC because it does not require the derivatization of non-volatile analytes, although both methods have been previously used for the analysis of metabolites from monolignol or lignan synthesis (Chen et al., 2017; Decembrino et al., 2020; Liu et al., 2017; Ricklefs et al., 2015). Currently described quantification methods comprise analysis of either hydroxycinnamic acids, -aldehydes, and -alcohols (Liu et al., 2017) or coniferyl alcohol and subsequent lignans (Decembrino et al., 2020, 2021). Separate quantification for both parts of the metabolic pathway prolongs analysis time and leads to an increased susceptibility to errors. The separation of the various substrates, intermediates, and products of the monolignol and lignan synthesis pathway in a single run is desired but challenging.

Reliable assessment of the metabolic state of cells requires analysis of intracellular titers (Oldiges et al., 2007). Extracellular metabolite titers might be distorted, causing a bias in quantification and thus hindering optimization. Previously published studies in the field of heterologous monolignol or lignan production analyzed combined samples of medium and cells, customarily extracted with ethyl acetate (Chen et al., 2017; Decembrino et al., 2020; Liu et al., 2017; Ricklefs et al., 2016; Zhang and Stephanopoulos, 2013). However, the effectiveness of extraction of intracellular metabolites with ethyl acetate has not been well investigated, and to our knowledge, no study analyzed extra- and intracellular monolignol and lignan titers separately. Quantification of intracellular metabolites requires their efficient release from cells without intrinsic modification or degradation of metabolites (Pinu et al., 2017). A specific method might be suitable for some classes of metabolites but inadequate for extraction of other classes (Duportet et al., 2012; Maharjan and Ferenci, 2003; Winder et al., 2008). For metabolic studies of microorganisms, chemical cell disruption methods are favored over mechanical or enzymatic disruption methods. A comparison of different chemical cell disruption methods is advisable to investigate the effect of solvent, temperature, and acidic/alkaline conditions on the metabolites of interest (Pinu et al., 2017).

In this work, we established a targeted metabolomics method for quantifying extra- and intracellular concentrations of metabolites from the monolignol and lignan synthesis pathway. To this end, an HPLC-DAD-MS protocol was developed and various methods for the extraction of intracellular metabolites were adapted and compared. As proof of concept, the applicability of the new method for pathway engineering and time-resolved monitoring of metabolite accumulation was validated.

## 2 Material and methods

### 2.1 Media

Media components were purchased from Carl Roth (Karlsruhe, Germany), Merck (Darmstadt, Germany), AppliChem (Darmstadt, Germany), Sigma-Aldrich (St. Louis, USA), and VWR International (Radnor, USA).

Bacterial cultures necessary during the cloning procedure were cultivated in lysogeny broth (LB) medium, containing 10.00 g L^−1^ tryptone, 5.00 g L^−1^ yeast extract, and 10.00 g L^−1^ NaCl. LB medium with 15.00 g L^−1^ agar was used for plate cultures. Precultures of *E. coli* strains type I (AK_RgTAL, AK_RsTAL, and AK_HaTAL, Fig. 1, Table S3) were prepared in M9 medium for BioLector® experiments or M9* medium for shaking flask experiments. M9 medium contained 5.00 g L^−1^ glucose, 8.50 g L^−1^ Na_2_HPO_4_, 3.00 g L^−1^ KH_2_PO_4_, 5.00 g L^−1^ NaCl, 10.00 g L^−1^ NH_4_Cl, 0.24 g L^−1^ MgSO_4_, and 1 mL L^−1^ trace stock solution. The trace stock solution was composed of 4.87 g L^−1^ FeSO_4_∙7 H_2_O, 4.12 g L^−1^ CaCl_2_∙2 H_2_O, 1.50 g L^−1^ MnCl_2_∙4 H_2_O, 1.87 g L^−1^ ZnSO_4_∙7 H_2_O, 0.30 g L^−1^ H_3_BO_3_, 0.25 g L^−1^ Na_2_MoO_4_∙2 H_2_O, 0.15 g L^−1^ CuCl_2_∙2 H_2_O, 0.84 g L^−1^ Na_2_EDTA∙2 H_2_O and 82.81 mL L^−1^ 37% HCl. With 25.50 g L^−1^ Na_2_HPO_4_ and 9.00 g L^−1^ KH_2_PO_4_, the concentration of phosphate salts was three times higher in M9*, but else of the same composition as M9. Main cultures for monolignol production with *E. coli* strains type I were grown in M9* medium, additionally supplemented with 0.40 g L^−1^ tyrosine. Pre- and main cultures for *E. coli* strains type II (ER_CgL1 and ER_CueO, Fig. 1, Table S3) were cultivated in a slightly modified Riesenberg medium containing 15.00 g L^−1^ glucose, 13.30 g L^−1^ KH_2_PO_4_, 4.00 g L^−1^ (NH_4_)_2_HPO_4_, 0.45 g L^−1^ MgSO_4_, and 2 mL L^−1^ trace stock solution. If necessary, 50 mg L^−1^ streptomycin, 50 mg L^−1^ kanamycin, or 100 mg L^−1^ ampicillin were added to the medium of plate-, pre- and main cultures for selection and plasmid stability. The initial pH of M9*, M9, and Riesenberg medium was adjusted to 7.0.

### 2.2 Recombinant DNA manipulation

Cloning and plasmid assembly were performed according to standard molecular biology methods (Green and Sambrook, 2012) and the methods described in 2.2.1, 2.2.2, 2.2.3, and 2.2.4. These methods were applied to assemble the various intermediary and final plasmids. Assembled vectors were transformed into *E. coli* DH5α after restriction and ligation cloning or *E. coli* One Shot™ TOP10 (Thermo Fisher Scientific Inc., Waltham, USA) for FastCloning. All plasmids used and/or constructed during this study are listed in Table S2.

#### 2.2.1 PCR for overhang addition

Polymerase chain reaction (PCR) was performed with GoTaq® DNA Polymerase (5 U μL^−1^) in 5X Green GoTaq reaction buffer (Promega, Madison, USA) according to the manufacturer's protocol. Primers used for PCR reactions are listed in Table S1. If necessary, overhangs with restriction sites were added via PCR. Amplified fragments were purified from the gel with the Wizard® SV Gel and PCR Clean-Up System (Promega) according to the manufacturer's protocol.

#### 2.2.2 Restriction, ligation, and transformation

PCR fragments and isolated plasmids were restricted in CutSmart® buffer. Restriction enzymes and buffers were purchased from New England Biolabs (Ipswich, USA). DNA (1–2 μg) was cut with 0.5–1 μL of enzyme. The restriction was performed for 1.5 h at 37 °C. For restriction of plasmids, 1 μL of calf intestinal phosphatase (CIP or Quick CIP, New England Biolabs) was added for dephosphorylation after the first incubation period, followed by a second incubation step at 37 °C for 30 min. Restricted inserts and backbones were purified from gel with the Wizard SV Gel and PCR Clean-Up System (Promega) according to the manufacturer's protocol. Ligation reaction mixtures contained T4 ligase reaction buffer, 1 μL T4 ligase (both acquired from Promega, 3 U μL^−1^) as well as cut and purified DNA in a ratio of at least a 1:1 (insert:vector), based on the band intensities of vector and insert after agarose gel electrophoresis. The ligation mix was filled up to 20 μL with ultrapure H_2_O and incubated at 16 °C overnight or 4 °C for three to four days. After this incubation period, DNA was precipitated by adding 1–2 μL glycogen and 65 μL 100% ethanol to the ligation reaction mixture. The supernatant was removed after centrifugation at 18,000*g* and 4 °C for 2–4 h. For resuspension of the DNA pellet, 250 μL of 70% (v/v) ethanol was used, prior to another centrifugation step at 18,000*g* and 4 °C for 40 min. The supernatant was discarded, the DNA pellet dried at 37 °C, and dissolved in 5 μL ultrapure H_2_O. The plasmid was transformed into *E. coli* DH5α via electroporation (Green and Sambrook, 2012).

#### 2.2.3 FastCloning and transformation

In 18 amplification cycles, the insert and vector were independently amplified, with overhangs of ∼16 bp added to both ends of the insert that were overlapping to the ends of the amplified vector (Li et al., 2011). Primers are listed in Table S1. The PCR mixtures were prepared with *Pfu* DNA polymerase (3 U μL^−1^) in *Pfu* reaction buffer with MgSO_4_ (Promega) according to the manufacturer's protocol. After the PCR reactions, 4 μL of insert DNA was merged with 4 μL of backbone DNA as well as 0.5 μL DpnI, followed by an incubation period of 1 h at 37 °C and subsequent inactivation at 80 °C for 10 min. The reaction mixture was used for transformation into chemically competent *E. coli* TOP10 cells.

#### 2.2.4 Verification of plasmid constructs

After transformation, colony PCR (cPCR) was performed to verify the correct insertion of the fragment into the plasmid backbone. According to the manufacturer's protocol, PCRs were run with primers listed in Table S1 and GoTaq DNA Polymerase (5 U μL^−1^) in Green GoTaq reaction buffer (Promega). Clones with inserts of the right size were cultivated overnight in 5 or 20 mL LB medium. Plasmids were isolated with the PureYield™ Plasmid Miniprep System (Promega) according to the manufacturer's protocol. Successful cloning was verified by DNA sequence analysis (Eurofins Genomics Germany, Ebersberg, Germany) using the primers listed in Table S1.

#### 2.2.5 Plasmid assembly

Cloning procedures were performed according to the methods described in 2.2.1, 2.2.2, and 2.2.3, using the restriction enzymes named in this section. These methods were applied to assemble the various intermediary and final plasmids as described below. All plasmids used and/or constructed during this study are listed in Table S2. Vector pRSFDuet_*At*CCoAOMT was constructed by ligating *at*ccoaomt, cut from template pUC57_*At*CCoAOMT (ordered from GENEWIZ, South Plainfield, USA), into the empty vector pRSFDuet-1 (Merck). Both template and empty vector were cut with restriction enzymes NcoI and HindIII before ligation. The construction of pETM6_*Xx*TAL_HpaBC(op) (*Xx* = *Rg*, *Ha*, or *Rs*) is based on various intermediary cloning vectors and pETM6_*Rg*TAL^syn^_HpaBC (Jones et al., 2017). Via FastCloning, *rgtal* and *hpaB* were amplified from pETM6_*Rg*TAL^syn^_HpaBC and inserted into pETM6, achieving vectors pETM6_*Rg*TAL and pETM6_HpaB. *hpaC* was ligated into backbone pRSFDuet after restriction sites were added via PCR with pETM6_*Rg*TAL^syn^_HpaBC as template and insert as well as backbone were cut with NdeI and XhoI, resulting in pRSFDuet_HpaC. Subsequently, the gene was cut from pRSFDuet_HpaC with NdeI and XhoI and inserted into pETM6, previously restricted with the same enzymes, obtaining pETM6_HpaC. *hpaB* and *hpaC* were assembled into an operon by ligating the *hpaC* insert, previously cut from pETM6_HpaC with XbaI and SalI, into the backbone pETM6_HpaB which was cut with SpeI and SalI, resulting in plasmid pETM6_HpaBC(op). Restriction sites for NdeI and KpnI were attached to *rstal* during PCR with plasmid pETDuet_*Rs*TAL as the template. The insert *rstal* and receiving backbone pETM6 were cut with these restriction enzymes, followed by ligation, achieving vector pETM6_*Rs*TAL. Cloning of plasmid pETM6_*Ha*TAL was carried out by amplifying *hatal* with an attached restriction site for KpnI from pCDFDuet_*Ha*TAL. The amplified insert and backbone pETM6 were restricted with enzymes XbaI and KpnI and subsequently ligated. Final working vectors pETM6_*Rg*TAL_HpaBC(op), pETM6_*Ha*TAL_HpaBC(op) and pETM6_*Rs*TAL_HpaBC(op) were obtained by ligation of insert hpaBC (cut from vector pETM6_HpaBC(op) with AvrII and SalI) and backbone pETM6_*Xx*TAL (previously cut with NheI and SalI).

### 2.3 Strain construction

The strains used in this study are listed in Table S3. Experiments for monolignol production were performed with *E. coli* BL21(DE3).G213 (G213), a proprietary strain from Phytowelt GreenTechnologies (Cologne, Germany) which contains two synthetic operons consisting of either *rstal*-*pc4cl* or *zmccr*-*zmcad*, both controlled by the same artificial constitutive promoter. G213 was used in combination with plasmids pRSFDuet_*At*CCoAOMT and pETM6_*Rg*TAL_HpaBC(op) (AK_RgTAL), pETM6_*Rs*TAL_HpaBC(op) (AK_*Rs*TAL) or pETM6_*Ha*TAL_HpaBC(op) (AK_HaTAL).

Experiments for lignan production were performed with *E. coli* OverExpress™ C43(DE3) (C43, Lucigen, Middleton, USA). *E. coli* strain ER_CueO corresponds to C43 harboring plasmid pCDFDuet_syfiPLR. C43 containing pCDFDuet_syfiPLR and pET16b_*CgL*1 is referred to as ER_CgL1. Both plasmids were previously published (Ricklefs et al., 2016).

### 2.4 Fermentation condition

#### 2.4.1 Long-term storage

Cells were stored at −80 °C as glycerol stocks with 17% glycerol for long-term storage. Cells from the glycerol stocks were spread on LB agar plates and grown at 37 °C overnight to obtain single colonies.

#### 2.4.2 Shaking flask cultivation

Precultures with 20 mL of medium were inoculated from plate cultures and grown overnight at 180–200 rpm and 30–37 °C. The precultures were used to inoculate 50–100 mL fresh medium in baffled shaking flasks (20% working volume) to an optical density at 600 nm (OD_600_) of 0.1. Main cultures were stirred at 180 rpm. When an OD_600_ of 0.6 was reached, 0.1–0.75 mM Isopropyl-β-D-thiogalactopyranoside (IPTG) was added to induce the expression of *lacO*-controlled promoters. Cultivation was stopped 24 h after induction. Main cultures were performed in duplicate.

AK_RgTAL was cultivated in M9* medium. Precultures were grown at 30 °C. Main cultures were supplemented with 0.4 g L^−1^ tyrosine at the start of cultivation and grown at 26 °C. 0.1 mM IPTG was added for induction at an OD_600_ of 0.6.

All shaking flask cultivations for lignan production with strains ER_CueO and ER_CgL1 were performed in the modified Riesenberg medium. Precultures were incubated at 37 °C. Main cultures were grown at 37 °C until induction, after which temperature was reduced to 30 °C. Cells were induced with 0.75 mM IPTG. At the same time, 0.5 g L^−1^ coniferyl alcohol and 50 μM CuSO_4_ were added to cultures as substrate and cofactor.

#### 2.4.3 BioLector cultivation

Precultures (20 mL M9 medium) were inoculated with colonies of AK_RsTAL, AK_HaTAL, or AK_RgTAL and incubated overnight at 30 °C, 180 rpm. Precultures were used to inoculate 1 mL of fresh M9* medium (supplemented with 0.4 g L^−1^ tyrosine) to an OD_600_ of 0.1. Strains were cultivated in FlowerPlates® (m2p labs, Baesweiler, Germany) at 26 °C and stirred at 1100 rpm in the BioLector microbioreactor (m2p labs). Cultures were induced with 0.5 mM IPTG at an approximate OD_600_ of 0.6. Cultivations were finished 19 h after induction and performed in duplicate.

#### 2.4.4 Determination of cell density

Cell density was determined via optical density. OD_600_ was measured with a spectrophotometer (Libra S11 Visible Spectrophotometer, Biochrom, Cambridge, UK). The cell dry weight concentration (CDW) was calculated from OD_600_. An OD_600_ of 1 corresponded to 0.312 g L^−1^ CDW.

### 2.5 Analysis of extra- and intracellular metabolites

#### 2.5.1 Sampling for intra- and extracellular metabolite analysis

Samples for extra- and intracellular metabolite analysis were either taken at the end of cultivation (comparison of enzymes/enzyme variants) or every 4 h after induction (time-resolved cultivation for monolignol or lignan synthesis). Cell pellets of 1.12 mg CDW were harvested for intracellular analysis. The only exceptions were BioLector experiments when fewer cells (800 μL of bacterial culture, ∼0.6 mg CDW) were collected due to the low working volume. The supernatant was removed using fast centrifugation (21,100*g*, 30 s, 4 °C), followed by quenching of cell metabolism via shock-frosting the cell pellet in liquid nitrogen. Cell pellets were stored at −20 °C until further use. Cell disruption and metabolite extraction was carried out using four different methods, which were adapted from literature (Maharjan and Ferenci, 2003; Teleki et al., 2015; Winder et al., 2008) and are described in 2.5.2, 2.5.3, 2.5.4, and 2.5.5. For extracellular metabolite analysis, samples of cell culture were centrifuged for 15 min at 21,100*g*, 4 °C for removal of cells. The supernatant was stored at −20 °C before quantification of extracellular carbon sources as well as monolignol and lignan metabolites via HPLC(-MS) analysis.

#### 2.5.2 Perchloric acid cell disruption (HClO_4_)

A volume of 100 μL HClO_4_ (0.25 M, 4 °C) was added to the cell pellet. Cells were resuspended by vortexing for 30 s. In three freeze-thaw cycles, the cell suspension was frozen in liquid nitrogen and thawed on ice. The cell debris was separated by centrifugation at 21,100*g* for 10 min (4 °C). The supernatant was transferred to a second tube and neutralized with 100 μL KOH (0.25 M, 4 °C). After brief vortexing, the precipitate was removed by centrifugation at 21,100*g* for 10 min (4 °C). The supernatant was transferred again to a new reaction tube and centrifuged for a further 20 min at 21,100*g* (4 °C) to pellet the remaining solid particles. The metabolite extract was subjected to HPLC-DAD-MS analysis.

#### 2.5.3 Hot water cell disruption (H_2_O)

Ultrapure water (100 μL, 4 °C) was added to the cell pellet. The mixture was directly incubated at 99 °C for 1 min in a ThermoMixer (Eppendorf, Hamburg, Germany), before vortexing for 30 s. Afterward, the cell suspension was again incubated at 99 °C for 5 min and then cooled on ice for 10 min. Most of the cell debris was removed by centrifugation at 21,100*g* for 10 min (4 °C). The supernatant was carried over to a new reaction tube and centrifuged again for 20 min at 21,100*g* (4 °C). The resulting metabolite extract was analyzed via HPLC-DAD-MS.

#### 2.5.4 Cold methanol cell disruption (MeOH)

The cell pellet was resuspended in 100 μL MeOH (LC-MS grade, −20 °C). It was subjected to three freeze-thaw cycles by freezing it in liquid nitrogen and allowing it to thaw on ice. Cell debris was pelleted by centrifugation at 21,100*g* for 10 min (4 °C). After removal, the supernatant was stored on ice. The pellet was resuspended again in 100 μL MeOH (LC-MS grade, −20 °C) and extracted via three freeze-thaw cycles with liquid nitrogen. Remaining debris was removed by centrifugation at 21,100*g* for 10 min (4 °C). The supernatant was pooled with the supernatant from the first cell disruption cycle and dried via vacuum centrifugation at 30 °C. The solid residue was dissolved in 100 μL ultrapure water. Afterward, undissolved particles were removed by centrifugation at 21,100*g* for 10 min (4 °C). The supernatant was transferred to a new reaction tube and subjected to a final centrifugation step at 21,100*g* for 20 min (4 °C). The metabolite extract was examined via HPLC-DAD-MS.

#### 2.5.5 Potassium hydroxide cell disruption (KOH)

Preheated KOH (100 μL, 0.25 M, 80 °C) was added to the cell pellet, which was resuspended by vortexing for 30 s. The cell suspension was incubated at 80 °C for 10 min and then cooled on ice for 10 min. Afterward, cell debris was removed by centrifugation at 21,100*g* for 10 min (4 °C). The supernatant was neutralized with 100 μL HClO_4_ (0.25 M, 4 °C). Precipitated KClO_4_ was separated by an additional centrifugation step at 21,100*g* for 10 min (4 °C). For further removal of remaining solid particles, final centrifugation was performed at 21,100*g* for 20 min (4 °C). Analysis of the metabolite extract occurred via HPLC-DAD-MS.

#### 2.5.6 Metabolite stability and extraction efficacy of disruption methods

All described cell disruption methods – HClO_4_, H_2_O, MeOH, and KOH – were compared concerning their effect on metabolite stability and extraction capability for intracellular metabolites. Stability was evaluated by subjecting 20 μL of an artificial metabolite mix (∼18 mg L^−1^ of each metabolite 1–4, 6–8, 10–12, and 14–20, see Table 1 and Fig. 1) to the procedure of the different cell disruption methods and comparing them to an untreated reference sample. This untreated reference sample was diluted with an equal volume of ultrapure H_2_O instead of solvents used during extraction and not subjected to the conditions applied during cell disruption with the various methods. Metabolite release from cells was assessed by applying all four cell disruption methods to cell pellets taken from the same culture at the same time (20 h after induction) and comparing all extracted metabolite titers. Extraction of metabolites of the monolignol pathway (3,4, 6–8, 10–12) was determined for a culture of AK_RgTAL. The release of metabolites of the lignan synthesis pathway (14–18) was analyzed for a culture of ER_CueO. Twelve samples of each culture were taken for intracellular metabolite analysis (three samples per cell disruption method). Experiments for the evaluation of metabolite stability and metabolite release were performed in triplicate.

**Table 1:** Commercial manufacturers of standards of monolignol and lignan metabolites. Metabolites are numbered according to Fig. 1.

#	Metabolite	Manufacturer
1	Phenylalanine	AppliChem (Darmstadt, Germany)
2	Cinnamic acid	Acros Organics (Geel, Belgium)
3	Tyrosine	Alfa Aesar (Haverhill, USA)
4	*p*-Coumaric acid	Sigma-Aldrich (St. Louis, USA)
5	*p*-Coumaryl-CoA	MicroCombiChem (Halsenbach, Germany)
6	*p*-Coumaryl aldehyde	Toronto Research Chemicals (Toronto, Canada)
7	*p*-Coumaryl alcohol	MicroCombiChem (Halsenbach, Germany)
8	Caffeic acid	Sigma-Aldrich (St. Louis, USA)
10	Caffeyl aldehyde	MicroCombiChem (Halsenbach, Germany)
11	Caffeyl alcohol	PhytoLab (Vestenbergsgreuth, Germany)
12	Ferulic acid	Acros Organics (Geel, Belgium)
14	Coniferyl aldehyde	Sigma-Aldrich (St. Louis, USA)
15	Coniferyl alcohol	Alfa Aesar (Haverhill, USA)
16	Pinoresinol	Carbosynth (Compton, United Kingdom)
17	Lariciresinol	Carbosynth (Compton, United Kingdom)
18	Secoisolariciresinol	Sigma-Aldrich (St. Louis, USA)
19	Matairesinol	Carbosynth (Compton, Great Britain)
20	Pluviatolide	Santa Cruz Biotechnology (Dallas, USA)

#### 2.5.7 Quantification of glucose concentration

Prior to analysis, samples were filtered with 0.45 μm polyamide filters (Macherey-Nagel, Düren, Germany). HPLC coupled to a refractive index (RI) detector (Agilent 1200 Series/1260 Infinity, Agilent Technologies, Santa Clara, USA) was used to quantify the glucose concentration. Separation was performed with a Metab-AAC column (Isera GmbH, Düren, Germany) (300 × 7.8 mm, 10 μm) and an isocratic flow of 0.5 mL min^−1^ 5 mM H_2_SO_4_. The column was kept at 40 °C and injections were performed every 30 min. Concentrations were calculated from peak areas of RI signals via an external calibration curve ranging from 0 to 15 g L^−1^ glucose.

#### 2.5.8 Quantification of monolignol and lignan metabolites

Standards of metabolites of the monolignol and lignan pathway were commercially acquired from the manufacturers listed in Table 1.

An artificial mix of metabolites 1–4, 6–8, 10–12, and 14–20, each with a concentration of ∼18 mg L^−1^, was used to develop the HPLC-DAD-MS protocol. Before analysis, samples of extracellular metabolites were filtered with 0.45 μm polyamide filters (Macherey-Nagel). Intra- and extracellular metabolites were identified and quantified via HPLC-DAD-MS. Identification of analytes was performed by comparing retention time with standards and mass spectrometry in positive ion mode. Separation of analytes was performed with a reversed-phase (RP) EC 100/2 Nucleoshell RP18 column (100 × 2 mm) with a particle size of 2.7 μm (Macherey-Nagel). Samples of most experiments were analyzed with a 1260 Infinity II LC System coupled to a 6120 quadrupole (Agilent, Santa Clara, USA), using the following gradient of 0.1% formic acid (A) and LC-MS grade methanol (B): 0–2 min 2% B, 2–4 min 2–25% B, 4–26 min 25–35% B, 26–29 min 35–90% B, 29–31 min 90%B, 31–32 min 90-2% B, 32–37 min, 2% B. The flow rate was kept at 0.3 mL min^−1^ and the column was tempered at 30 °C. For MS settings, a capillary voltage of 3000 V was combined with a drying gas flow of 12 L min^-1^ at 350 °C and a nebulizer pressure of 2.41 bar. For analysis of BioLector experiments, another 1260 Infinity II LC System (Agilent, Santa Clara, USA) coupled to a compact QTOF (Bruker, Billerica, USA) was used with a slightly modified gradient at a flow rate of 0.3 mL min^−1^ and 30 °C: 0–2 min 2% B, 2–4 min 2–25% B, 4–25 min 25–35% B, 25–26 min 35–90% B, 26–31 min 90% B, 31–32 min 90–2% B, 32–37 min 2% B. The compact QTOF analysis was performed with a capillary voltage of 4500 V, a drying gas flow of 12 L min^−1^ (220 °C), and a nebulizer pressure of 4 bar. For all experiments, analytes were quantified via UV absorption between 200 and 340 nm with a DAD (see also Supplementary Table S4), using external calibration curves of standards. The presence of metabolites 1–4, 6–8, 10–12, and 14–20 within samples was verified via mass spectrometry. If a metabolite-specific mass peak was not detected and thus no peak was observed in the extracted ion chromatogram (EIC, see Table S4) at the expected retention time, the peak area of the UV detection was not integrated.

#### 2.5.9 Calculation of intracellular metabolite concentration

Intracellular metabolite titers (c_intra_) were calculated by multiplying the metabolite concentration measured during HPLC-DAD-MS analysis (c_HPLC_) with a dilution factor. This factor is based on the dilution of the absolute intracellular volume with the solvent used during cell disruption (V_solvent_). The absolute intracellular volume is calculated from the CDW at the sampling time, the sample volume (V_sample_), and the specific intracellular volume (SIV). According to a study estimating the intracellular volume of *E. coli* BL21(DE3), an SIV of 1.9 μL mg^−1^_CDW_ was assumed to determine intracellular metabolite concentrations (L. Wang et al., 2013).

$$c_{intra}=\;c_{HPLC}\;\times \left(\;\frac{V_{solvent}+\left(CDW\;\times \;V_{sample}\;\times \;SIV\right)\;}{CDW\;\times \;V_{sample}\;\times \;SIV}\right)$$

## 3 Results

### 3.1 Development of a quantification method for the monolignol and lignan synthesis pathway

For developing a chromatographic method separating all required monolignol and lignan metabolites, an RP18 column was chosen due to the low polarity of the majority of the compounds. To properly separate the polar substrate tyrosine (**3**) from the void volume and thus the injection peak, a low portion (2%) of the polar organic solvent MeOH was chosen as starting condition to facilitate the interaction of tyrosine with the stationary C18 phase. The quick increase in organic solvent from 2 to 25% MeOH followed by a very flat slope decreases analysis time while enabling segregation and elution of less polar molecules with rather similar structures. A steep increase in the MeOH portion towards the end of the method is intended to remove non-polar, previously not eluted compounds from the column. During the last few minutes, the starting conditions are reset for re-equilibration of the column.

**Fig. 2:**
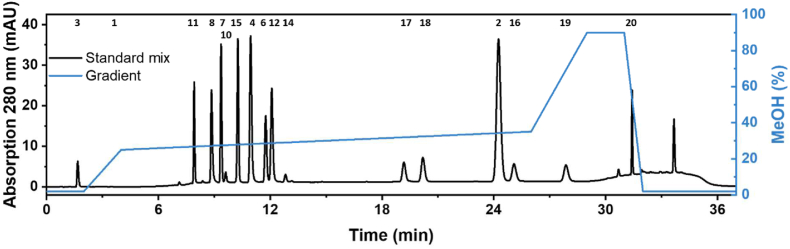
Separation of monolignol and lignan metabolites from phenylalanine/tyrosine to pluviatolide. DAD measurement at 280 nm during RP18 HPLC separation of an artificial metabolite standard mix containing metabolites 1–4, 6–8, 10–12,14–20, ∼18 mg L^−1^ each (Table 1) is displayed. A gradient of MeOH and 0.1% formic acid was used at a flow rate of 0.3 ml min^−1^. Peaks are numbered according to Fig. 1. Phenylalanine (1) is only represented by a number in this chromatogram, as it does not exhibit sufficient absorption at 280 nm.

These requisites were converted to an HPLC protocol and tested for the separation and identification of metabolites of an artificial mixture of commercially available metabolite standards (Fig. 2). For all metabolites except phenylalanine (**1**), peaks were visible in the UV chromatogram at 280 nm. Phenylalanine exhibits no absorption at 280 nm (Wetlaufer, 1963) and was detected at 200 nm instead. Due to different UV absorption maxima of the various analytes, absorption at wavelengths between 200 and 340 nm was used for quantification (see Table S4). All metabolites in the mix were sufficiently separated, with resolutions of at least 1.5 of each peak to the previous peak, which is considered as baseline separation (Seidel-Morgenstern, 2020). Chromatographic and mass spectrometry parameters such as retention time, resolution, most prominent mass-to-charge ratio, and linear range as well as coefficient of determination of external UV standard curves, are listed in Table S4. The standard of *p*-coumaryl-CoA (**5**) was neither detected by UV absorption nor mass spectrometry (data not shown). Standards of caffeyl-CoA (**9**) and feruloyl-CoA (**13**) were not acquired. Thus, CoA thioesters are not quantifiable with the newly developed method. Nevertheless, this method is well suited for the separation and detection of all other metabolites of the monolignol and lignan synthesis pathway from aromatic amino acids to pluviatolide.

### 3.2 Selection of an optimal extraction method for monolignol and lignan metabolites

**Fig. 3:**
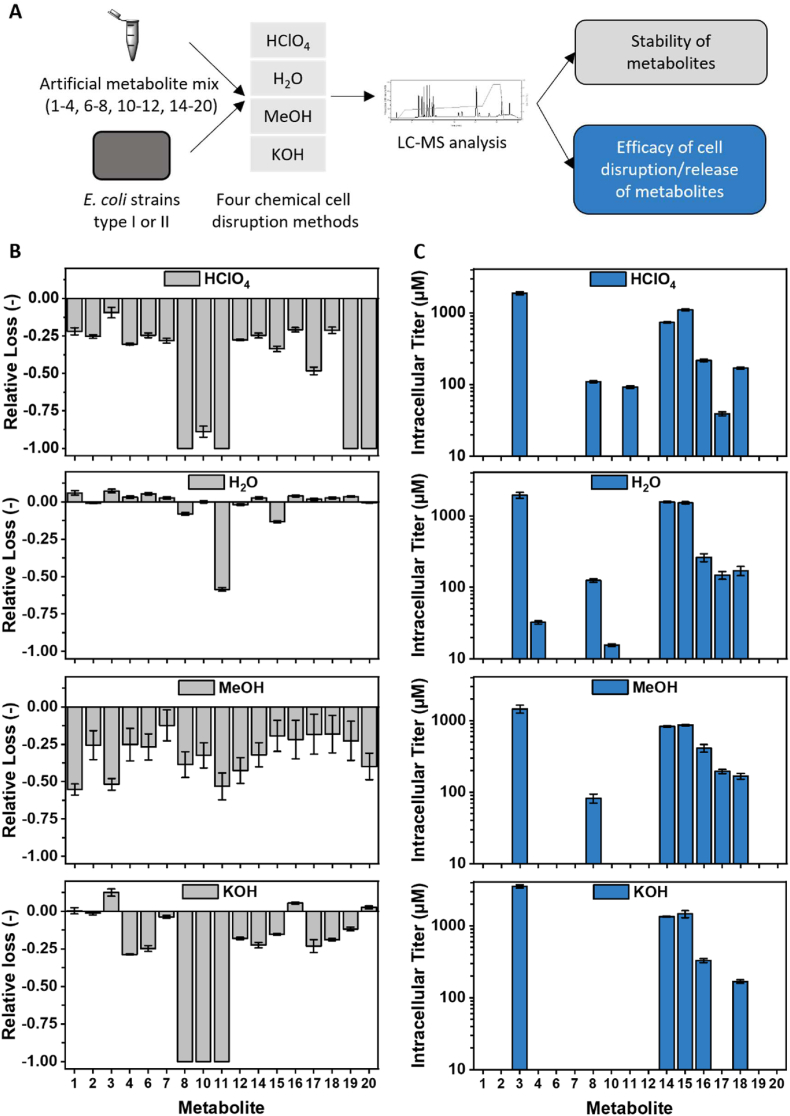
Evaluation of cell disruption methods. (A) Perchloric acid (HClO_4_), hot water (H_2_O), cold methanol (MeOH), and potassium hydroxide (KOH) cell disruption methods were applied to (B) an artificial metabolite mix (containing metabolites 1–4, 6–8, 10–12, 14–20, ∼18 mg L^−1^ each, Table 1) as well as (C) to cell pellets of a strain producing either coniferyl alcohol or secoisolariciresinol. (B) Relative loss of metabolite concentration in metabolite mix after treatment with various cell disruption procedures. (C) Determined intracellular metabolite concentrations after cell disruption with various methods. The intracellular concentration of metabolites 1–4, 6–8, and 10–12 was determined for cell pellets of AK_RgTAL. Cell pellets of ER_CueO were used to assess the intracellular concentration of metabolites 14–20. The numbering of metabolites corresponds to Fig. 1.

With the HPLC-DAD-MS method described above, all substrates, intermediates, and (by)products of the lignan synthesis pathway are quantifiable, except CoA thioesters. Extracellular titers, which are commonly analyzed, do not necessarily reflect intracellular metabolite titers reliably. Intracellular metabolite analysis is thus advisable, but the choice of extraction method might also negatively affect the accurate quantification of titers. Four chemical cell disruption methods – HClO_4_, H_2_O, MeOH, and KOH – were compared concerning metabolite stability and extraction efficacy to determine which of the tested methods is best suited for the extraction of metabolites of the monolignol and lignan synthesis pathway (Fig. 3A). To evaluate metabolite stability, an artificial mix of metabolites 1–4, 6–8, 10–12, and 14–20 (∼18 mg L^−1^ each) was subjected to the procedures of cell disruption. For assessment of extraction efficacy, samples of the same culture were disrupted with all four methods. To this end, the cell pellets of two different recombinant strains producing either coniferyl alcohol from tyrosine (AK_RgTAL) or secoisolariciresinol from coniferyl alcohol (ER_CueO) were used. The corresponding extracellular titers at the time of cell harvest are displayed in Fig. S1.

Regarding metabolite stability (Fig. 3B), 3-hydroxylated metabolites (caffeic acid (**8**), caffeyl aldehyde (**10**), and caffeyl alcohol (**11**)) were least stable under acidic and alkaline conditions. Matairesinol (**19**) and pluviatolide (**20**) were also prone to acidic degradation but more stable in an alkaline environment. Almost all other metabolites were also sensitive against the addition of acid or base, although to a lesser degree. Few metabolites were unstable in water at a high temperature. Caffeyl alcohol (**11**) was least stable during H_2_O extraction, with a loss of approx. 60% of the initial concentration. Caffeic acid (**8**) and coniferyl alcohol (**15**) also showed reduced stability when treated with hot water, recovering approx. at 90% of the initial concentration. For some metabolites, treatment with hot water led to an increase in the concentration of maximal 7%. An increase of up to 12% was also observed for a few metabolites after the application of the KOH cell disruption procedure. Presumably, this increase in concentrations is due to the evaporation of water during the incubation period at 99 (H_2_O) or 80 °C (KOH). After applying the MeOH cell disruption method to the metabolite mix, losses between 12 and 55% were perceived for all metabolites of interest. Since this is not due to high temperature during extraction or the use of acidic/alkaline solvents, it might result from decreased solubility or degradation during vacuum centrifugation. All in all, the highest stability of monolignol and lignan metabolites of the artificial mix was observed for the H_2_O cell disruption method.

The necessary enzymes for the synthesis of cinnamic acid (**2**), ferulic acid (**12**), matairesinol (**19**), and pluviatolide (**20**) were not included in the strains used for the assessment of extraction efficacy (Fig. 1). Thus, these analytes were not detected in intracellular samples (Fig. 3C). Additionally, phenylalanine (**1**), *p*-coumaryl aldehyde (**6**), and *p*-coumaryl alcohol (**7**) were not observed in intracellular samples. However, these metabolites were also not detected in the extracellular supernatant and probably did not accumulate sufficiently (Fig. S1). Accordingly, the evaluation of extraction efficacy is based on the other metabolites. Intracellular caffeyl alcohol (**11**) was only quantifiable after sample treatment with HClO_4_. Caffeic acid (**8**) was measured in roughly the same concentration in samples of cell pellets disrupted with HClO_4_, H_2_O, and MeOH. Both caffeic acid (**8**) and caffeyl alcohol (**11**) were previously shown to be unstable when acidic or alkaline solvents were directly applied to the artificial metabolite mix. Degradation of these metabolites appears less distinct in the presence of cells, probably due to a pH buffering effect of the latter. H_2_O was the only method that enabled the quantification of *p*-coumaric acid (**4**) and caffeyl aldehyde (**10**). Lariciresinol (**17**) was challenging to extract from cell pellets with HClO_4_ and KOH. With a strain producing *p*-coumaryl alcohol (**7**) instead of coniferyl alcohol (**15**), intracellular *p*-coumaric acid (**4**), *p*-coumaryl aldehyde (**6**), and *p*-coumaryl alcohol (**7**) were measured in all samples after cell disruption with either HClO_4_, H_2_O, MeOH, or KOH, although the titers differed vastly (data not shown). Of the tested cell disruption methods, H_2_O enabled the extraction of the highest number of metabolites and resulted in the highest intracellular titers measured for most of the metabolites.

In summary, among the various methods tested, H_2_O cell disruption was best suited for extracting intracellular monolignol and lignan metabolites with reference to metabolite stability and extraction efficacy. Consequently, extraction with hot water was employed to analyze intracellular metabolites in further experiments.

### 3.3 Scrutinizing the developed targeted metabolomics method with challenging pathway reactions

**Fig. 4:**
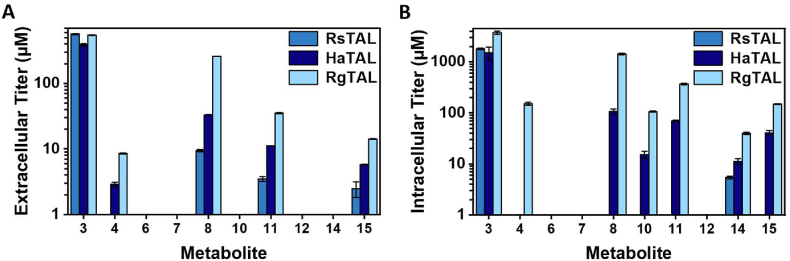
Metabolic profiling of strains AK_RsTAL, AK_HaTAL, and AK_RgTAL. (A) Extracellular monolignol metabolite titers at 19 h after induction with IPTG. (B) Intracellular monolignol titers at 19 h after induction. The numbering of metabolites corresponds to Fig. 1.

**Fig. 5:**
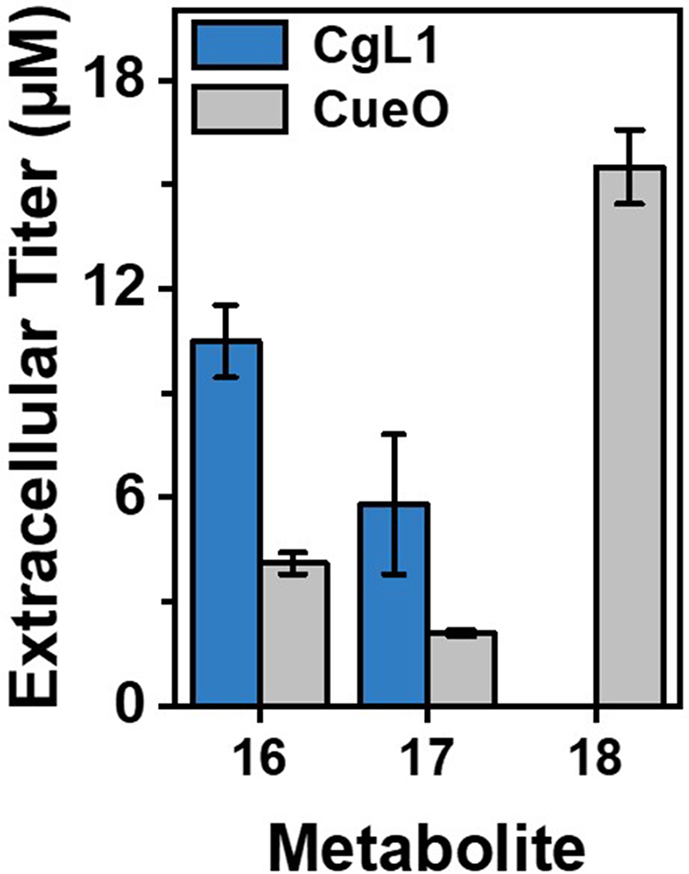
Comparison of lignan production by strains ER_CgL1 and ER_CueO. Extracellular lignan titers were measured 24 h after induction with IPTG. Dimerization of coniferyl alcohol was initiated by one-electron oxidation catalyzed by CgL1 or CueO. The numbering of metabolites corresponds to Fig. 1.

To prove the suitability of the targeted metabolomics methods for pathway engineering aiming to optimize monolignol and lignan synthesis, we analyzed and compared the pathway activity of strains expressing different enzymes catalyzing reactions which are known to be challenging. These reactions are the tyrosine deamination catalyzed by a TAL and the one-electron oxidation initiating coniferyl alcohol coupling to pinoresinol, for instance, catalyzed by a laccase. For the first bottleneck, deamination of tyrosine, three different TAL variants (*Rs*TAL from *Rhodobacter sphaeroides*, *Ha*TAL from *Herpetosiphon aurantiacus*, or *Rg*TAL from *Rhodotorula glutinis*) were compared regarding their impact on coniferyl alcohol production and the overall accumulation of intermediates and (by)products (Fig. 4). For the second challenging reaction, one-electron oxidation of coniferyl alcohol, the effect on the accumulation of pinoresinol and subsequent lignans was investigated for the heterologous laccase *Cg*L1 from *Corynebacterium glutamicum* and the endogenous multicopper oxidase CueO of *E. coli* (Fig. 5). The corresponding final cell dry weights of these cultivations are displayed in Figs. S2 and S3.

The expression of the different TALs substantially affected the accumulation of intermediates and (by)products. The highest extracellular titers of coniferyl alcohol (**15**) and other metabolites of the monolignol pathway were measured with the strain expressing *Rg*TAL (14.2 μM coniferyl alcohol), which was 5.7 and 2.5 times higher than for AK_RsTAL (2.5 μM) and AK_HaTAL (5.8 μM), respectively (Fig. 4A). Correspondingly, intracellular accumulation of coniferyl alcohol was 3.7 times higher when expressing *Rg*TAL (148.6 μM) instead of *Ha*TAL (40.6 μM) (Fig. 4B). Except for tyrosine (**3**) and coniferyl aldehyde (**14**), no intracellular metabolite titers were measured in extracted intracellular samples of AK_RsTAL. Extracellular accumulation of caffeic acid (**8**), the most abundant extracellular intermediate, was 27 and 8 times higher in the supernatant of the strain expressing *Rg*TAL (257.8 μM) compared to the expression of *Rs*TAL (9.4 μM) and *Ha*TAL (33.1 μM), respectively. Within cells, caffeic acid was also the highest accumulating intracellular intermediate, reaching titers of 106.4 μM (AK_HaTAL) and 1432.2 μM (AK_RgTAL). Accumulation of caffeyl alcohol (**11**) was also observable for all strains, except in intracellular samples of AK_RsTAL. Opposing extra- and intracellular metabolite titers, extracellular titers were roughly one magnitude lower than intracellular titers. Aldehydes (**6**, **10**, **14**) appear to primarily remain within the cells since they were measured in extracted samples of cells but not in the supernatant.

Concerning the second bottleneck, coupling of coniferyl alcohol, the heterologous expression of *Cg*L1 resulted in higher extracellular accumulation of pinoresinol ((**16**), 10.5 μM) and lariciresinol ((**17**), 5.8 μM) compared to the ER_CueO strain with 4.1 and 2.1 μM, respectively (Fig. 5). However, secoisolariciresinol (**18**), the final product of the cascade, was only measured in extracellular samples of ER_CueO with a titer of 15.5 μM. Since (−)-secoisolariciresinol is a product of the PLR reaction with (+)-pinoresinol as substrate and (+)-lariciresinol as intermediate, more pinoresinol was formed by the ER_CueO strain.

In conclusion, different enzymes/enzyme variants catalyzing the deamination of tyrosine (TALs) or the one-electron oxidation of coniferyl alcohol (laccases) substantially impacted the accumulation of subsequent intermediates and products. The strains AK_RgTAL and ER_CueO exhibited the highest titers of their respective final products coniferyl alcohol and secoisolariciresinol. Using our newly developed method, we were able to show the effect of two different enzymes not only on their reactants but also on other measured metabolites. The results demonstrate the potential of the developed targeted metabolomics method for monolignol and lignan pathway engineering.

### 3.4 Time-resolved analysis of extra- and intracellular monolignol and lignan accumulation

We intended to monitor the development of extra- and intracellular monolignol and lignan titers more closely throughout the cultivation, to investigate whether different phases or temporary maxima of metabolite accumulation occur. The strains AK_RgTAL and ER_CueO were cultivated for the production of coniferyl alcohol (**15**) or secoisolariciresinol (**18**), respectively. Samples for extra- and intracellular metabolite analysis were taken every 4 h after induction.

**Fig. 6:**
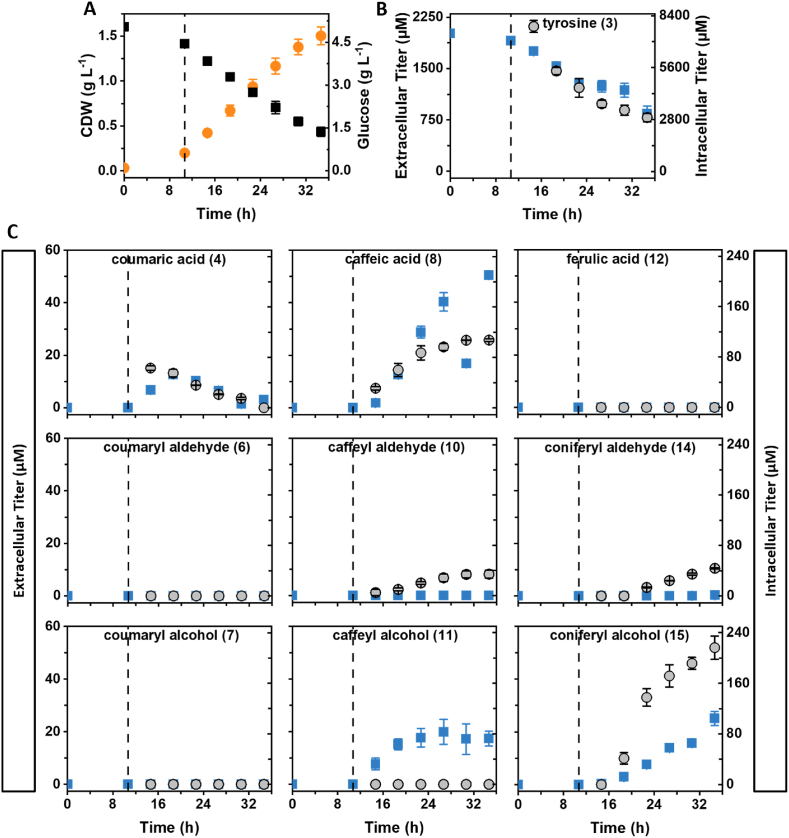
Tracking of extra- and intracellular monolignol metabolites during cultivation of strain AK_RgTAL. A dashed line indicates the time of induction. (A) CDW (orange circles) and glucose consumption (black squares). (B) Extra- (blue squares) and intracellular (grey circles) tyrosine titers over time. (C) Extra- (blue squares) and intracellular (grey circles) monolignol metabolite titers over time. The numbering of metabolites corresponds to Fig. 1. (For interpretation of the references to color in this figure legend, the reader is referred to the Web version of this article.)

For strain AK_RgTAL producing coniferyl alcohol (**15**) from tyrosine (**3**), this time-resolved analysis showed a constant decrease of the extra- and intracellular tyrosine titers (Fig. 6). The subsequent metabolite of the cascade, *p*-coumaric acid (**4**), accumulated both extra- and intracellularly during the first few hours after induction and decreased again over the course of the cultivation. In contrast, caffeic acid (**8**) accumulation continued throughout cultivation, although the increase in intracellular concentration flattened towards the end of cultivation. In accordance with our previous results, caffeyl aldehyde (**10**) and coniferyl aldehyde (**14**) were not detected in the culture supernatant. However, increasing aldehyde titers were observed intracellularly. An increase in the extracellular caffeyl alcohol (**11**) titer was observed during the first 8 h after induction. In contrast, caffeyl alcohol was not measured in intracellular samples, probably due to thermal degradation during the hot water extraction process (Fig. 3). After induction, constant coniferyl alcohol (**15**) accumulation was observed both within and outside the cells. No extra- or intracellular accumulation was observed for *p*-coumaryl aldehyde (**6**), *p*-coumaryl alcohol (**7**), and ferulic acid (**12**).

**Fig. 7:**
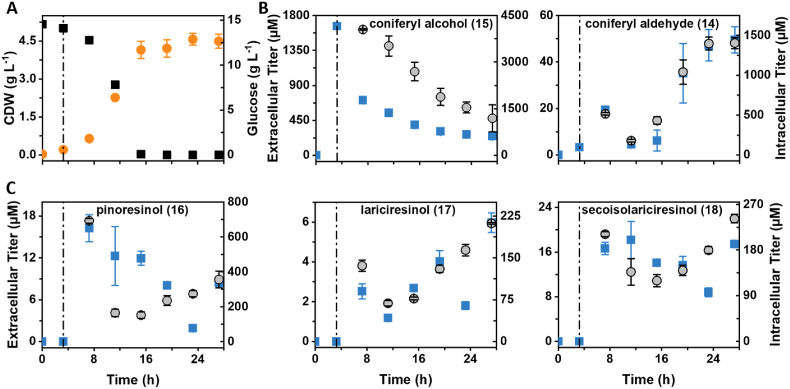
Monitoring of extra- and intracellular lignan metabolite titers over time of strain ER_CueO. A pointed-dashed line indicates the time of induction as well as the addition of coniferyl alcohol and CuSO_4_. (A) CDW (orange circles) and glucose consumption (black squares). (B) Extra- (blue squares) and intracellular (grey circles) coniferyl alcohol and – aldehyde titers over time. (C) Extra- (blue squares) and intracellular (grey circles) lignan titers over time. The numbering of metabolites corresponds to Fig. 1. (For interpretation of the references to color in this figure legend, the reader is referred to the Web version of this article.)

For the ER_CueO culture producing secoisolariciresinol (**18**) from coniferyl alcohol (**15**), a quick decrease of the substrate concentration in the medium was observed within the first 4 h after addition. Within cells, the coniferyl alcohol concentration decreased more constantly over time (Fig. 7). Extra- and intracellular coniferyl aldehyde (**14**) accumulation reached a temporary maximum at 4 h after induction. The titers in both sample types decreased temporarily before accumulation continued during the stationary phase. The same trend was also observed for intracellular titers of pinoresinol (**16**), lariciresinol (**17**), and secoisolariciresinol (**18**), with the lowest titers measured during the late exponential phase at 11–15 h cultivation time (8–12 h after induction). In contrast, no clear tendency was discernible for the progression of extracellular titers of the same metabolites. After induction, pinoresinol (**16**), lariciresinol (**17**), and secoisolariciresinol (**18**) were measured in the supernatant, but titers fluctuated throughout the cultivation.

Overall, we showed that during the monitored cultivation time, accumulation occurred constantly for some metabolites, whereas transient maxima were observed for other metabolites. Our developed targeted metabolomics method is applicable for the sensitive time-resolved analysis of pathway activity. The described differences of titers in dependence on cultivation duration demonstrate the importance of metabolite monitoring for assessing pathway activity and bottleneck identification. Additionally, they emphasize the shortcomings of one-point measurements.

## 4 Discussion

### 4.1 Developing a targeted metabolomics method for extra- and intracellular monolignol and lignan analysis

The synthesis pathway for heterologous microbial production of complex lignans from inexpensive substrates contains multiple potential bottlenecks and junction points for byproduct formation. Determination of the metabolic state facilitates strain and process optimization (Oldiges et al., 2007), but previously published quantification methods comprise only sections of the monolignol and lignan pathway and do not consider intracellular metabolite pools separately. Thus, we engaged ourselves in developing a comprehensive chromatographic quantification method and optimizing the extraction of intracellular monolignol and lignan metabolites.

To quantify as many substrates, intermediates, and (by)products as possible, we successfully established an HPLC method. It is capable of separating all commercially available metabolites of the monolignol and lignan synthesis pathway between tyrosine (**3**)/phenylalanine (**1**) and pluviatolide (**20**), except CoA thioesters (**5**,**9**,**13**) (Fig. 2). Hydroxycinnamyl-CoA thioesters are more polar than tyrosine. Therefore, their separation requires the use of ion-pairing reagents (Obel and Scheller, 2000) or a hydrophilic interaction liquid chromatography (HILIC) column (Qualley et al., 2012). However, the accumulation of CoA thioesters is unlikely due to their labile nature, and they are commonly not quantified in studies of microbial monolignol production.

For the analysis of intracellular metabolites, we pursued a method that permitted the most accurate reflection of the metabolic state within cells, since the stability and extractability of different metabolites depend on the method used for cell disruption. Studies analyzing intracellular pools of tyrosine and phenylalanine (Taymaz-Nikerel et al., 2009), *p*-coumaric acid (Barnhart-Dailey et al., 2019), cinnamic acid, and the lignan-related polyphenols stilbenes were published (van Summeren-Wesenhagen and Marienhagen, 2015; Zhao et al., 2018). However, no method for the extraction of further monolignols or lignans is currently available. Thus, we compared different chemical cell disruption methods, covering various parameters such as incubation temperature, the polarity of the solvent used, the application of acidic/alkaline conditions, and the application of freeze-thaw cycles. As expected, the stability and extraction efficacy of individual metabolites differed vastly between the different methods (Fig. 3). In previous comparative studies of cell disruption methods, extraction with cold methanol achieved the most comprehensive analysis of the global intracellular metabolome of microorganisms (Maharjan and Ferenci, 2003; Villas-Bôas et al., 2005; Winder et al., 2008). However, the highest stability and extraction efficacy of metabolites of interest was observed after extraction with hot water in this study. Hot water is less frequently used in metabolome studies than HClO_4_ or MeOH, but achieved near-complete extraction of metabolites, comparable to that of cold methanol, in previous studies (Canelas et al., 2009; Park et al., 2012). Additionally, H_2_O was reported to be advantageous in regard to reproducibility (Hiller et al., 2007). H_2_O cell disruption is not well suited for the extraction of thermolabile or highly concentrated non-polar metabolites. However, it is easy to execute and compatible with MS-based analytics.

The purposefulness of intracellular metabolite analysis is demonstrated by the data obtained for the development of lignan titers over cultivation time (Fig. 7). No clear trend was observed for extracellular titers of pinoresinol (**16**), lariciresinol (**17**) and secoisolariciresinol (**18**). In contrast, intracellular titers exhibited a clear trend. Fluctuation of extracellular lignan titers might be a result of low stability outside the cells or oxidation followed by oligo-/polymerization (Decembrino et al., 2021).

When regarding the depletion of the substrate (tyrosine or coniferyl alcohol) and the accumulation of intermediates and (by)products (Fig. 6, Fig. 7), it is obvious that the masses are not balanced. However, this is probably not due to an error in quantification of the respective metabolites, but to the occurrence of further unquantified byproducts. For instance, tyrosine as a proteinogenic amino acid is also incorporated into proteins. Lignans might be oxidized, generating lignan oligo-/polymers (Tarrago et al., 2018). Furthermore, radical coupling of coniferyl alcohol is known to result in the formation of coupling byproducts like dehydrodiconiferyl alcohol, amongst others (Pickel and Schaller, 2013). Due to the unavailability of commercial standards, these byproducts were not quantified and are thus missing in the mass balance. This demonstrates a limitation of the targeted approach of the newly developed metabolomics method of this study.

### 4.2 Comprehensive analysis of metabolite levels enables pathway optimization

The developed method is applicable for metabolic engineering, as it enables analysis of the effect of different enzymes/enzyme variants on all metabolites of the whole pathway. This might be crucial for the correct assessment of pathway activity. For instance, extracellular pinoresinol (**16**) titers were lower for samples of ER_CueO cultures with an endogenous multicopper oxidase compared to the strain expressing the heterologous laccase *Cg*L1, thereby falsely indicating lower activity (Fig. 5). However, when subsequent metabolites are considered, it becomes clear that more pinoresinol must have been synthesized in the ER_CueO strain, as has been previously reported by Decembrino et al. The produced pinoresinol was partly used as substrate by syfiPLR, but a portion of (±)-pinoresinol was probably further oxidized and subsequently oligo-/polymerized (Decembrino et al., 2021).

Another argument emphasizing the importance of measuring as many metabolites as possible is that the measurement of the final product alone might lead to underestimation of pathway activity due to the emergence of unexpected bottlenecks or byproducts. For example, the intracellular titer of the final product coniferyl alcohol (**15**) was 3.7 times higher when *Rg*TAL was expressed instead of *Ha*TAL. In comparison, the intracellular caffeic acid (**8**) titer was 13.5 times higher in samples of AK_RgTAL cultures than in those of AK_HaTAL, since CoA-ligation of caffeic acid (**8**) became a second bottleneck. Thus, analysis of all measurable intermediates and byproducts is reasonable, as it facilitates the identification of bottlenecks. The mentioned bottleneck of CoA ligation is also observable in the constant accumulation of caffeic acid during time-resolved analysis (Fig. 6). Hydroxylation of coumaric acid appears to be a transient bottleneck. Due to its constitutive promoter, the genomic copy of *rstal* is expressed earlier than *hpaBC*, the gene of 4HPA3H, which is under the control of the IPTG-inducible T7 promoter. Therefore, temporary accumulation of coumaric acid is observed before it is depleted by 4HPA3H after induction. The formation of caffeyl alcohol indicates the 3-OH methylation of caffeyl-CoA as another bottleneck in the pathway towards coniferyl alcohol. Accumulation of intermediates or byproducts occurs if the reaction rates throughout the pathway are not adjusted, for instance, due to variation of expression, in vivo activities, or half-lives of the involved enzymes. As presented in our work, this might be overcome by using alternative enzymes catalyzing the same reaction. Further options to adjust reaction rates and ensure an optimal flow of carbon from the substrate to the product are the modification of protein activity and stability via protein engineering (Li et al., 2020), and the optimization of gene copy numbers and promoter strengths (Jones and Koffas, 2016). Additionally, improvement of cofactor availability might be beneficial (Y. Wang et al., 2013).

Consistent with previous publications, both CoA ligation by 4CL (Rodrigues and Rodrigues, 2021) and 3-OH methylation by CCoAOMT (Chen et al., 2017) were determined as additional bottlenecks. The results obtained for TAL comparison are also in accordance with previously published studies, in which both RgTAL (Santos et al., 2011) and HaTAL (Jendresen et al., 2015) exhibited higher activity than RsTAL. Thus, the newly established targeted metabolomics method enables the reproduction of results obtained in previous studies. It is suitable to compare different enzyme variants to alleviate bottlenecks and enables the detection of temporal changes in metabolite pools.

## 5 Conclusion

Until now, a concise method for analyzing heterologously produced extra- and intracellular metabolites of the monolignol and lignan synthesis pathway was unavailable. In this study, we developed a short and comprehensive LC-MS method for the separation, identification, and quantification of these metabolites, omitting time-consuming and error-prone fragmented analysis approaches. Various cell disruption methods were compared concerning their capability to extract monolignol and lignan metabolites from *E. coli* cells. In addition, the potential bias imposed by the various extraction methods was investigated. Hot water cell disruption revealed a bias towards underestimating intracellular caffeyl alcohol titers but was otherwise best suited respecting extraction efficacy and metabolite stability of those metabolites of interest. The combinatorial cell disruption of split cell samples with two complementary methods, for instance, H_2_O and HClO_4_, could further improve the completeness and accuracy of metabolite extraction. To demonstrate its applicability, the newly developed targeted metabolomics method was used to compare different enzyme variants for alleviation of bottlenecks and monitor metabolite titers in close sampling intervals. It will promote further strain and process development studies and facilitate pathway optimization.

## Author contributions

Andrea Steinmann: Conceptualization, Methodology, Validation, Formal Analysis, Investigation, Data Curation, Writing-Original Draft, Writing – Review & Editing, Visualization.

Katrin Schullehner: Conceptualization, Resources, Writing- Review & Editing, Project Administration.

Anna Kohl: Conceptualization, Methodology, Formal Analysis, Investigation, Resources, Writing – Review & Editing.

Christina Dickmeis: Conceptualization, Methodology, Formal Analysis, Investigation, Resources, Writing – Review & Editing.

Maurice Finger: Conceptualization, Methodology, Validation, Investigation, Writing – Review & Editing.

Georg Hubmann: Conceptualization, Writing – Review & Editing, Supervision.

Guido Jach: Conceptualization, Resources, Writing – Review & Editing, Supervision, Project Administration, Funding Acquisition.

Ulrich Commandeur: Conceptualization, Resources, Writing – Review & Editing, Supervision, Project Administration, Funding Acquisition.

Marco Girhard: Conceptualization, Resources, Writing – Review & Editing, Supervision, Project Administration, Funding Acquisition.

Vlada B. Urlacher: Conceptualization, Resources, Writing – Review & Editing, Supervision, Project Administration, Funding Acquisition.

Stephan Lütz: Conceptualization, Data Curation, Writing – Review & Editing, Supervision, Project Administration, Funding Acquisition.

## Declaration of competing interest

The authors declare that they have no known competing financial interests or personal relationships that could have appeared to influence the work reported in this paper.
