# Supplementary material for: A targeted metabolomics method for extra- and intracellular metabolite quantification covering the complete monolignol and lignan synthesis pathway
Source: Metab Eng Commun. 2022 Aug 31;15:e00205. doi: 10.1016/j.mec.2022.e00205 (PMC9474286; doi:10.1016/j.mec.2022.e00205)
Supplement: Multimedia component 1 [file mmc1.docx]

**Supplementary data**

Table S1: Primers used for cloning and sequencing.

| **Primer name** | **Sequence (5'→3')** | **Application** |
| --- | --- | --- |
| M1_*Rg*TAL_fw | GAAGGAGATATACATATGGCGCCTCGCCCGAC | FastCloning (*rgtal*) |
| M1_*Rg*TAL_rv | GGTTTCTTTACCAGACTCGAGTTATGCCAGCATCTTC | FastCloning (*rgtal*) |
| M1_pETM6_fw | CTGAAGATGCTGGCATAACTCGAGTCTGGTAAAG | FastCloning (*rgtal*) |
| M1_pETM6_rv | GTCGGGCGAGGCGCCATATGTATATCTCCTTC | FastCloning (*rgtal*) |
| M2_HpaB_fw | GAAGGAGATATACATATGAAACCAGAAGATTTC | FastCloning (*hpaB*) |
| M2_HpaB_rv | CTTTACCAGACTCGAGTTATTTCAGCAGCTTATCCAG | FastCloning (*hpaB*) |
| M2_pETM6_fw | GATAAGCTGCTGAAATAACTCGAGTCTGGTAAAGAAAC | FastCloning (*hpaB*) |
| M2_pETM6_rv | GGAAATCTTCTGGTTTCATATGTATATCTCCTTCTTAAAG | FastCloning (*hpaB*) |
| 9_NdeI_HpaC_fw | GGAGATATACATATGCAATTAGATG | PCR (*hpaC*) |
| 10_HpaC_XhoI_rv | CTTTACCAGACTCGAGTTAAATCGCAGCTTCC | PCR (*hpaC*) |
| NdeI_*Rs*TAL_fw | ATACATATGAGCGCGCAGGATCCGGCTCTGGC | PCR (*rstal*) |
| *Rs*TAL_KpnI_rv | AGACTCGAGGGTACCTTAAACTGGACTCTGTTGCAGC | PCR (*rstal*) |
| DuetMCS1_fw | ATGCGTCCGGCGTAGA | PCR (*hatal*) |
| *Ha*TAL_KpnI_rv | AGACTCGAGGGTACCTTAGCGAAACAGAATAATACTACGC | PCR (*hatal*) |
| ACYCDuet UP1 | GGATCTCGACGCTCTCCCT | cPCR, sequencing |
| BamHI_*Ha*TAL_fw | AGCCAGGATCCGGGCAGCACCACCCTGATTCTG | cPCR, sequencing |
| MCS1_rv | GATTATGCGGCCGTGTACAA | cPCR, sequencing |
| pETM6_rv | GCTACAGGGCGCGTCCCATTCGCCAATCC | cPCR, sequencing |
| *Rg*TAL_end_fw | GCATTTGTGCGTGAGGAACTGGGTGTC | cPCR, sequencing |
| *Rs*TAL_end_fw | CTCATCTGCTGCAACAGAGTCC | cPCR, sequencing |
| pETM6_fw | ATTGTGAGCGGATAACAATTC | cPCR, sequencing |
| DuetMCS2_rv | GCTAGTTATTGCTCAGCGG | cPCR, sequencing |

Table S2: Plasmids used for cloning and monolignol or lignan production. op=operon, co=codon-optimized, Kan^R^=kanamycin-resistant, Amp^R^=ampicillin-resistant, Strep^R^=streptomycin-resistant.

| **Plasmid** | **Application** | **Selection** | **Reference** |
| --- | --- | --- | --- |
| pRSFDuet-1 | Empty vector | Kan^R^ | Novagen, Merck |
| pUC57_*At*CCoAOMT | Template for *atccoaomt* | Amp^R^ | GENEWIZ Inc.  Genbank AF360317.1 (co) |
| pRSFDuet_*At*CCoAOMT | Monolignol production | Kan^R^ | This work |
| pETM6 | Empty vector | Amp^R^ | Addgene plasmid # 49795  (Xu et al., 2012) |
| pETM6_*Rg*TAL^syn^_HpaBC | Template for *rgtal*, *hpaB*, *hpaC* | Amp^R^ | Addgene plasmid  #100949  (Jones et al., 2017) |
| pETDuet_*Rs*TAL | Template for *rstal* | Amp^R^ | (Jansen et al., 2014) |
| pCDFDuet_*Ha*TAL | Template for *hatal* | Strep^R^ | (Jendresen et al., 2015) |
| pETM6_*Rg*TAL | Intermediary cloning vector | Amp^R^ | This work |
| pETM6_HpaB | Intermediary cloning vector | Amp^R^ | This work |
| pRSFDuet_HpaC | Intermediary cloning vector | Kan^R^ | This work |
| pETM6_HpaC | Intermediary cloning vector | Amp^R^ | This work |
| pETM6_HpaBC(op) | Intermediary cloning vector | Amp^R^ | This work |
| pETM6_*Rg*TAL_HpaBC(op) | Monolignol production | Amp^R^ | This work |
| pETM6_*Ha*TAL | Intermediary cloning vector | Amp^R^ | This work |
| pETM6_*Ha*TAL_HpaBC(op) | Monolignol production | Amp^R^ | This work |
| pETM6_*Rs*TAL | Intermediary cloning vector | Amp^R^ | This work |
| pETM6_*Rs*TAL_HpaBC(op) | Monolignol production | Amp^R^ | This work |
| pCDFDuet_syfiPLR | Lignan production | Strep^R^ | (Ricklefs et al., 2016) |
| pET16b_*Cg*L1 | Lignan production | Amp^R^ | (Ricklefs et al., 2016) |

Table S3: Strains used in this study. ci=chromosome integrated, op= operon, Kan^R^=kanamycin resistance, Amp^R^=ampicillin resistance, Strep^R^=streptomycin resistance.

| Type | Name | Strain background | Plasmid(s) | Genetic modification | Selection | Application | Reference |
| --- | --- | --- | --- | --- | --- | --- | --- |
| - | DH5α | *E. coli* DH5α | - | - | - | Cloning procedure | Thermo Fisher  Scientific |
| - | TOP10 | *E. coli* TOP10 | - | - | - | Cloning procedure | Thermo Fisher  Scientific |
| - | BL21(DE3) | *E. coli* BL21(DE3) | - | - | - | Host | (Studier & Moffatt,  1986) |
| - | G213 | *E. coli* BL21(DE3).G213 | - | - | - | Host for monolignol  production | Phytowelt  GreenTechnologies  GmbH  (proprietary) |
| I | AK_RgTAL | *E. coli* BL21(DE3).G213 | pRSFDuet_*At*CCoAOMT  pETM6_*Rg*TAL_HpaBC(op) | pT7-*atccoaomt*  pT7-*rgtal*  pT7-*hpaBC*(op) | Kan^R^  Amp^R^ | Evaluation of cell  disruption methods  Comparison of TAL  variants  Time-resolved production  of coniferyl alcohol | This work |
| I | AK_RsTAL | *E. coli* BL21(DE3).G213 | pRSFDuet_*At*CCoAOMT  pETM6_*Rs*TAL_HpaBC(op) | pT7-*atccoaomt*  pT7-*rstal*  pT7-*hpaBC(op)* | Kan^R^  Amp^R^ | Comparison of TAL  variants | This work |
| I | AK_HaTAL | *E. coli* BL21(DE3).G213 | pRSFDuet_*At*CCoAOMT  pETM6_*Ha*TAL_HpaBC(op) | pT7-*atccoaomt*  pT7-*hatal*  pT7-*hpaBC*(op) | Kan^R^  Amp^R^ | Comparison of TAL  variants | This work |
| - | C43(DE3) | *E. coli* C43(DE3) | - | - | - | Host for lignan  production | Lucigen  (Miroux and  Walker, 1996) |
| II | ER_CueO | *E. coli* C43(DE3) | pCDFDuet_syfiPLR | pT7-*plr* | Strep^R^ | Evaluation of cell  disruption methods  Comparison of enzyme  variants  Time-resolved production  of secoisolariciresinol | This work |
| II | ER_CgL1 | *E. coli* C43(DE3) | pCDFDuet_syfiPLR  pET16b_*Cg*L1 | pT7-*plr*  pT7-*cgl1* | Strep^R^  Amp^R^ | Comparison of enzyme  variants | This work |

Table S4: Parameters for chromatographic and mass-spectrometric identification and quantification. Retention times and resolution correspond to a flow rate of 0.3 mL min^-1^.

| Analyte | | UV detection (nm) | Retention time t_R_ (min) | Resolution to previous analyte R (-) | Molecular weight (g mol^-1^) | Most prominent m/z (-) | MS adduct/  fragment | Linear range UV quantification (µM) | Coefficient of determination R^2^ |
| --- | --- | --- | --- | --- | --- | --- | --- | --- | --- |
| 1 | Phenylalanine | 200 | 3.4 | 5.7 | 165.2 | 166.1 | [M+H]^+^ | 0-605 | 0.9999 |
| 2 | Cinnamic acid | 280 | 24.3 | 10.7 | 148.2 | 149.1 | [M+H]^+^ | 0-675 | 1.0000 |
| 3 | Tyrosine | 280 | 1.7 | - | 181.2 | 182.1 | [M+H]^+^ | 0-552 | 1.0000 |
| 4 | *p*-Coumaric acid | 310 | 11.0 | 4.2 | 164.2 | 165.1 | [M+H]^+^ | 0-609 | 0.9998 |
| 5 | *p*-Coumaryl-CoA | NA | NA | NA | 913.7 | NA | NA | NA | NA |
| 6 | *p*-Coumaryl aldehyde | 310 | 11.8 | 4.3 | 148.2 | 149.1 | [M+H]^+^ | 0-405 | 1.0000 |
| 7 | *p*-Coumaryl alcohol | 260 | 9.4 | 3.8 | 150.2 | 133.1 | [M+H–H_2_O]^+^ | 0-399 | 0.9994 |
| 8 | Caffeic acid | 310 | 8.9 | 7.5 | 180.2 | 181.0 | [M+H]^+^ | 0-555 | 1.0000 |
| 9 | Caffeyl-CoA | NA | NA | NA | 929.7 | NA | NA | NA | NA |
| 10 | Caffeyl aldehyde | 340 | 9.6 | 5.4 | 164.2 | 165.0 | [M+H]^+^ | 0-365 | 0.9999 |
| 11 | Caffeyl alcohol | 260 | 7.9 | 25.1 | 166.2 | 149.1 | [M+H–H_2_O]^+^ | 0-361 | 0.9996 |
| 12 | Ferulic acid | 325 | 12.1 | 1.7 | 194.2 | 195.1 | [M+H]^+^ | 0-1545 | 0.9995 |
| 13 | Feruloyl-CoA | NA | NA | NA | 943.7 | NA | NA | NA | NA |
| 14 | Coniferyl aldehyde | 340 | 12.8 | 3.7 | 178.2 | 179.1 | [M+H]^+^ | 0-515 | 0.9998 |
| 15 | Coniferyl alcohol | 260/280 | 10.3 | 4.5 | 180.2 | 163.0 | [M+H–H_2_O]^+^ | 0-555 | 0.9998 |
| 16 | Pinoresinol | 280 | 25.1 | 1.9 | 358.4 | 381.1 | [M+Na]^+^ | 0-279 | 1.0000 |
| 17 | Lariciresinol | 280 | 19.2 | 23.8 | 360.4 | 383.1 | [M+Na]^+^ | 0-277 | 1.0000 |
| 18 | Secoisolariciresinol | 280 | 20.2 | 3.0 | 362.4 | 327.1 | [M+H-2 H_2_O]^+^ | 0-276 | 1.0000 |
| 19 | Matairesinol | 280 | 27.9 | 6.7 | 358.4 | 359.1 | [M+H]^+^ | 0-837 | 1.0000 |
| 20 | Pluviatolide | 280 | 31.4 | 13.4 | 356.4 | 379.1 | [M+Na]^+^ | 0-84 | 0.9999 |


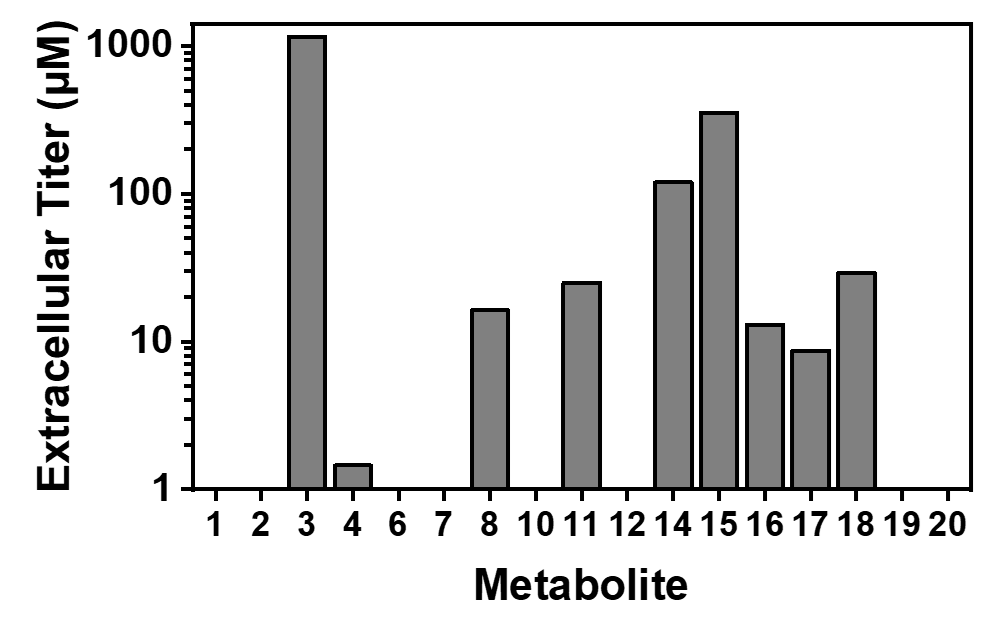


Figure S1: Extracellular metabolite titers of cultures used for evaluation of extraction efficacy at the time of cell harvest (20 h after induction). Extracellular concentration of metabolites 1–4, 6–8, and 10–12 was determined for a culture of AK_RgTAL. Extracellular concentration of metabolites 14–20 was determined for a culture of ER_CueO. The numbering of metabolites corresponds to Figure 1.

.


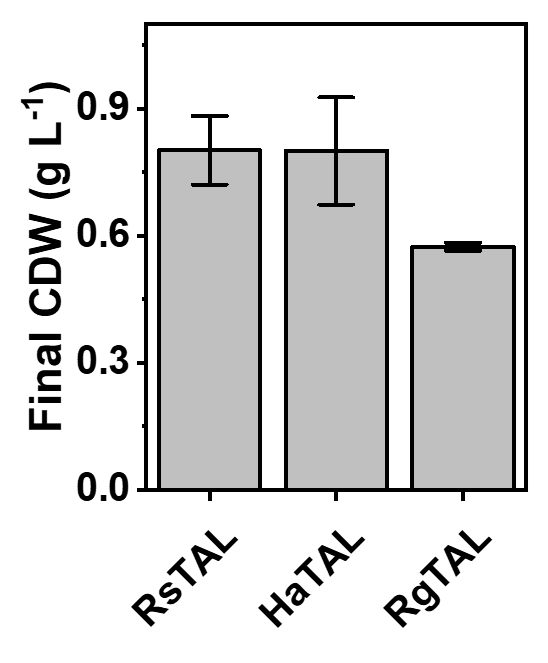


Figure S2: Final CDWs of strains AK_RsTAL, AK_HaTAL, and AK_RgTAL at 19 h after induction with IPTG.


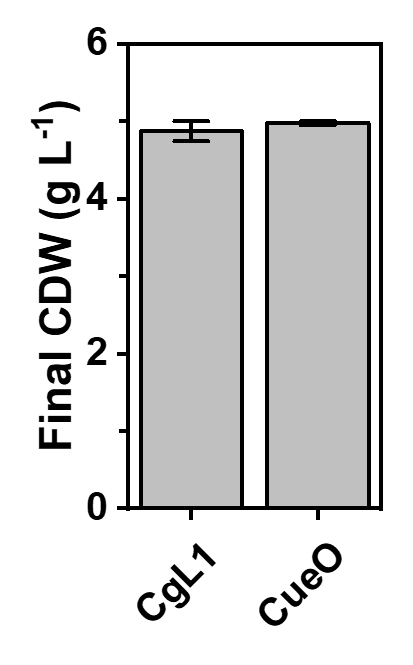


Figure S3: Final CDWs of strains ER_CgL1 and ER_CueO at 24 h after induction with IPTG.

**Literature**

Jansen, F., Gillessen, B., Mueller, F., Commandeur, U., Fischer, R., Kreuzaler, F., 2014. Metabolic engineering for p-coumaryl alcohol production in *Escherichia coli* by introducing an artificial phenylpropanoid pathway. Biotechnol. Appl. Biochem. 61, 646–654. https://doi.org/10.1002/bab.1222

Jendresen, C.B., Stahlhut, S.G., Li, M., Gaspar, P., Siedler, S., Förster, J., Maury, J., Borodina, I., Nielsen, A.T., 2015. Highly active and specific tyrosine ammonia-lyases from diverse origins enable enhanced production of aromatic compounds in bacteria and *Saccharomyces cerevisiae*. Appl. Environ. Microbiol. 81, 4458–4476. https://doi.org/10.1128/AEM.00405-15

Jones, J.A., Vernacchio, V.R., Collins, S.M., Shirke, A.N., Xiu, Y., Englaender, J.A., Cress, B.F., 2017. Complete Biosynthesis of Anthocyanins. MBio 8, 1–9.

Miroux, B., Walker, J.E., 1996. Over-production of proteins in *Escherichia coli*: Mutant hosts that allow synthesis of some membrane proteins and globular proteins at high levels. J. Mol. Biol. 260, 289–298. https://doi.org/10.1006/jmbi.1996.0399

Ricklefs, E., Girhard, M., Urlacher, V.B., 2016. Three-steps in one-pot: Whole-cell biocatalytic synthesis of enantiopure (+)- and (-)-pinoresinol via kinetic resolution. Microb. Cell Fact. 15, 1–11. https://doi.org/10.1186/s12934-016-0472-0

Studier, F.W., Moffatt, B.A., 1986. Use of bacteriophage T7 RNA polymerase to direct selective high-level expression of cloned genes. J. Mol. Biol. 189, 113–130. https://doi.org/10.1016/0022-2836(86)90385-2

Xu, P., Vansiri, A., Bhan, N., Koffas, M.A.G., 2012. EPathBrick: A synthetic biology platform for engineering metabolic pathways in *E. coli*. ACS Synth. Biol. 1, 256–266. https://doi.org/10.1021/sb300016b
